# Niche-mediated BMP/SMAD signaling regulates lung alveolar stem cell proliferation and differentiation

**DOI:** 10.1242/dev.163014

**Published:** 2018-05-11

**Authors:** Mei-I Chung, Melissa Bujnis, Christina E. Barkauskas, Yoshihiko Kobayashi, Brigid L. M. Hogan

**Affiliations:** 1 Department of Cell Biology, Duke University Medical School, Durham, NC 27710, USA; 2 Division of Pulmonary, Allergy and Critical Care Medicine, Department of Medicine, Duke University Medical School, Durham, NC 27710, USA

**Keywords:** Lung, Alveolar epithelium, AT1, AT2, BMP, Smad1/5/8, Follistatin, Noggin, Regeneration, Compensatory regrowth, Pneumonectomy

## Abstract

The bone morphogenetic protein (BMP) signaling pathway, including antagonists, functions in lung development and regeneration of tracheal epithelium from basal stem cells. Here, we explore its role in the alveolar region, where type 2 epithelial cells (AT2s) and Pdgfrα^+^ type 2-associated stromal cells (TASCs) are components of the stem cell niche. We use organoids and *in vivo* alveolar regrowth after pneumonectomy (PNX) – a process that requires proliferation of AT2s and differentiation into type 1 cells (AT1s). BMP signaling is active in AT2s and TASCs, transiently declines post-PNX in association with upregulation of antagonists, and is restored during differentiation of AT2s to AT1s. In organoids, BMP4 inhibits AT2 proliferation, whereas antagonists (follistatin, noggin) promote AT2 self-renewal at the expense of differentiation. Gain- and loss-of-function genetic manipulation reveals that reduced BMP signaling in AT2s after PNX allows self-renewal but reduces differentiation; conversely, increased BMP signaling promotes AT1 formation. Constitutive BMP signaling in Pdgfrα^+^ cells reduces their AT2 support function, both after PNX and in organoid culture. Our data reveal multiple cell-type-specific roles for BMP signaling during alveolar regeneration.

## Abstract (precis)

**Summary:** Studies using alveolar organoids and *in vivo* alveolar regrowth post-pneumonectomy establish many dynamic and cell-type-specific roles for BMP signaling in regulating adult alveolar regeneration.

## INTRODUCTION

The lung is a complex organ comprising a highly branched system of air-conducting tubes terminating in millions of air-exchanging units called alveoli. The alveolar epithelium is composed of two distinct cell types: type 1 and type 2 cells. Type 1 alveolar epithelial cells (AT1s) are very large, thin squamous cells that cover about 95% of the internal surface of the lung and are important for gas exchange between the air and blood in the capillaries. Type 2 alveolar epithelial cells (AT2s) are cuboidal and characterized by the production and secretion of pulmonary surfactant, preventing lung collapse during exhalation.

At steady state, alveolar cell turnover is low. However, efficient repair and regeneration has been reported following cellular damage or increased functional demand, both in animal models (see below) and humans (Butler et al., 2012; Kumar et al., 2011; Toufen et al., 2011). Adult AT2s, as a population, have been recognized as alveolar stem/progenitor cells capable of both self-renewal and differentiation into AT1s (Barkauskas et al., 2013; Desai et al., 2014; Evans et al., 1973; Hogan et al., 2014; Kapanci et al., 1969; Rock et al., 2011). The microenvironment in which AT2s reside encompasses a number of different cell types, including AT1s, Pdgfrα^+^ and Pdgfrβ^+^ stromal cells, endothelial cells, and immune cells. A number of studies have explored roles for these different components in models of lung repair and regeneration (Barkauskas et al., 2013; Chen et al., 2012; Ding et al., 2011; Jain et al., 2015a; Lechner et al., 2017; Lee et al., 2014; Liu et al., 2015, 2016; Nabhan et al., 2018; Rafii et al., 2015; Zacharias et al., 2018; Zepp et al., 2017). One such model is alveolar regrowth after pneumonectomy (PNX): the surgical removal of one or more lung lobes. This procedure, in different species, leads to compensatory regrowth of the remaining lung tissue, with formation of new blood vessels, epithelial and mesenchymal cells, and alveolar septa in order to restore alveolar number and surface area. In murine lungs, regrowth is achieved by 21 days after surgery, with the peak of AT2 proliferation occurring at around 7 days (Buhain and Brody, 1973; Butler et al., 2012; Green et al., 2016; Hsia et al., 1994; Thane et al., 2014).

So far, several factors and signaling pathways have been identified that promote the proliferation of AT2s after PNX. These include mechanical tension-induced YAP activation, EGF-related peptides released from the extracellular matrix by metalloproteinases secreted by endothelial cells in response to platelet-derived SDF1 signaling, and paracrine signals from activated macrophages (Ding et al., 2011; Lechner et al., 2017; Liu et al., 2016; Rafii et al., 2015). Compensatory regrowth involves not only increased proliferation of AT2s but also formation of new AT1s to restore alveolar surface area and pulmonary function. This conclusion is supported by the fact that a reduction in both AT2 proliferation and AT2 to AT1 differentiation is associated with impaired compensatory regrowth in the absence of YAP or activated macrophages (Lechner et al., 2017; Liu et al., 2016). One outstanding issue relevant to the biology of alveolar regrowth is the identity of all of the niche factors that promote AT2 cell proliferation and differentiation, and the cells producing and receiving them.

Here, we use both 3D organoid culture and *in vivo* studies to examine the role of BMP signaling in the AT2 stem cell niche. We find that post-PNX, Smad-dependent BMP signaling is transiently reduced in both AT2s and the Pdgfrα^+^ cells adjacent to them [referred to here as TASCs (type 2-associated stromal cells)]. This modulation involves changes in both BMP receptor levels and the upregulation of genes encoding BMP antagonists. Gain- and loss-of-function genetic manipulation *in vivo* reveals that loss of BMP signaling in AT2s after PNX allows their self-renewal but significantly reduces their ability to give rise to AT1s; conversely, increased BMP signaling promotes AT1 differentiation. Focusing on the contribution of the stroma to AT2 behavior, we provide evidence that they are a source of BMP antagonists and that constitutive BMP signaling in Pdgfrα^+^ fibroblasts reduces the ability of these cells to support AT2 proliferation, both *in vivo* and *in vitro*. Taken together, our studies thus establish multiple, dynamic, cell-type-specific roles for BMP signaling in regulating adult alveolar regeneration.

## RESULTS

### Dynamic BMP signaling in the AT2 niche during alveolar regrowth *in vivo*

**Fig. 1.**
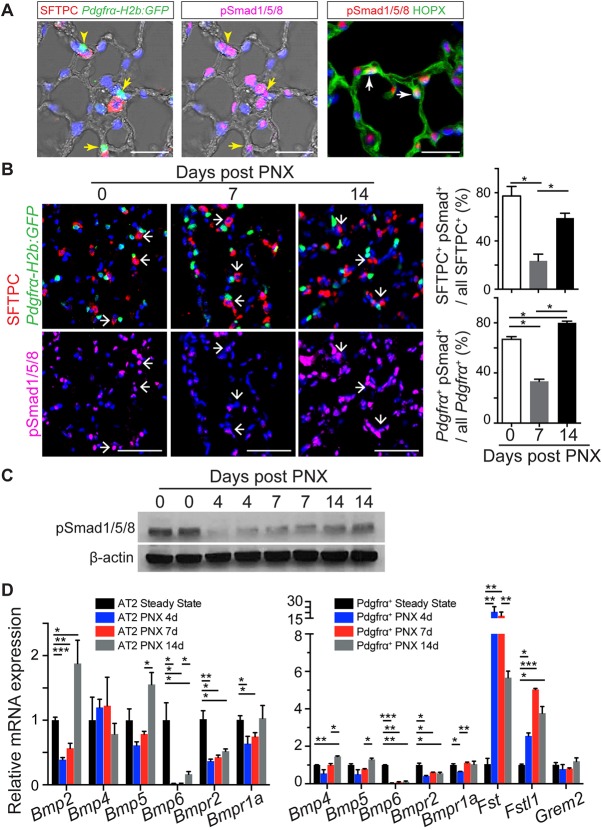
**Dynamic BMP signaling in the AT2 niche during alveolar regrowth *in vivo*.** (A) Expression of phospho-Smad1/5/8 (pSmad1/5/8), SFTPC, Pdgfrα-H2b:GFP and HOPX in the alveolar region of the adult mouse lung, as revealed by immunofluorescence and confocal microscopy. Left: SFTPC, H2b:GFP and nuclei (DAPI). Middle: same field of view, exposed to show pSmad1/5/8 and DAPI. Yellow arrows indicate Pdgfrα^+^ cells that are pSmad1/5/8^+^, while the yellow arrowheads indicate a Pdgfrα^+^ cell that is pSmad1/5/8^−^. Right: HOPX is both cytoplasmic and nuclear. White arrows indicate pSmad1/5/8^+^ AT1s. Scale bars: 20 μm. (B) Expression of pSmad1/5/8 (magenta) in SFTPC^+^ cells (red) and Pdgfrα-H2b:GFP^+^ cells in the alveolar region at steady state and 7 and 14 days after PNX. Arrows indicate AT2s adjacent to Pdgfrα^+^ TASCs. Scale bars: 50 μm. (C) pSmad1/5/8 levels in extracts of lungs after PNX. β-Actin is loading control. (D) qRT-PCR for transcripts of BMP signaling genes in lineage-labeled AT2s (left) and Pdgfrα-H2b:GFP stromal cells (right) at steady state and at times after PNX. The mRNA expression levels of PNX samples were normalized to steady state. Data are mean±s.e.m. *P<0.05, **P<0.01, ***P<0.001.

To explore whether BMP signaling plays a role in regulating lung alveolar regrowth after PNX, we examined canonical BMP signaling before and after removing the left lobe. At steady state, immunofluorescence analysis for phospho-Smad1/5/8 (pSmad1/5/8) shows that BMP signaling is active in many alveolar cells, including AT2s (77.9±0.8% of SFTPC^+^ cells) and Pdgfrα-H2B:GFP^+^ TASCs adjacent to them (66.9±2.1%) (Fig. 1A). Confocal microscopy shows that these TASCs have a characteristic morphology, with long cellular extensions (Fig. S1 and Movie 1). pSmad1/5/8 expression was also seen in AT1s (85.0±1.5% of HOPX^+^ cells), endomucin^+^ endothelial cells (64.5±0.9%) and Pdgfrb^+^ alveolar stromal cells (46.2±0.9%) (Fig. S2).

At 7 days post-PNX, around the peak of AT2 proliferation (Brody et al., 1978; Ding et al., 2011), the number of pSmad1/5/8^+^ AT2s was significantly reduced (Fig. 1B). However, by 2 weeks post-PNX, when proliferation is minimal and AT2s are robustly differentiating into AT1s (see Fig. 5 and Fig. S3), the proportion of pSmad1/5/8^+^ AT2s has returned closer to steady state levels (Fig. 1B). A similar result was seen in Pdgfrα^+^ TASCs in which the level of pSmad1/5/8^+^ expression was reduced on day 7 and restored on day 14 (Fig. 1B). A small reduction in pSmad1/5/8 was also seen in endomucin^+^ endothelial cells but no significant change was observed in either Pdgfrb^+^ stromal cells or AT1 cells (Fig. S2). The dynamic change in overall BMP signaling was confirmed using western blot analysis of pSmad1/5/8 levels in whole-lung lysates (Fig. 1C).

To better understand the mechanisms underlying the transient change in BMP signaling, qPCR analysis was used to follow the differential expression of pathway components, including ligands, receptors and antagonists. As shown in Fig. 1D, the expression of *Bmp6* and *Bmpr2* was significantly reduced in AT2s on days 4, 7 and 14 post-PNX, while *Bmp2* and *Bmpr1a* levels were reduced on days 4 and 7. A similar trend was also seen in the expression of *Bmp6* and *Bmpr2* in Pdgfrα^+^ cells. Significantly, transcripts encoding BMP antagonists, including follistatin (*Fst*) and follistatin-like 1 (*Fstl1*), were strongly upregulated in Pdgfrα^+^ cells. Some increase in the low levels of *Grem1* transcripts was detected (Fig. S2) but there was no apparent change in the expression of *Grem2* (which encodes an antagonist implicated by others in promoting AT2 growth (Zepp et al., 2017) (Fig. 1D).

### Pharmacological modulation of BMP signaling alters AT2 proliferation and differentiation in 3D organoid cultures

**Fig. 2.**
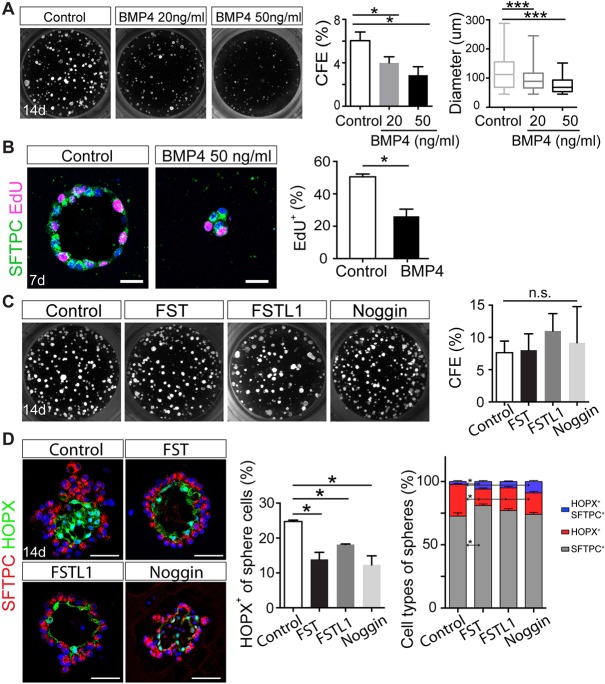
**Effect of BMP ligands and antagonists on AT2 cell proliferation and differentiation in 3D organoid culture.** (A) Left three panels: typical day 14 alveolosphere cultures, with and without BMP4. Graphs quantitate the effect of BMP4 on CFE and organoid size. (B) Effect of 50 ng/ml BMP4 on proliferation of SFTPC^+^ cells in spheres at 7 days, as judged by incorporation of EdU over a 2 h period. Scale bars: 20 μm. (C) Day 14 spheres cultured with BMP antagonists FST and FSTL1 (500 ng/ml) and Noggin (1 μg/ml). No significant difference in CFE was seen. (D) Immunofluorescence analysis of SFTPC^+^ (AT2s) and HOPX^+^ (AT1s) revealed a reduction in AT2 to AT1 differentiation in spheres exposed to different BMP antagonists for 14 days. Left graph shows the percentage of total cells in multiple spheres that are HOPX^+^. Right graph shows the percentage of total cells that are SFTPC^+^, HOPX^+^, and SFTPC^+^ HOPX^+^, as judged by immunofluorescence of sections. For all experiments, *n*=3 animals. Data are mean±s.e.m. *P<0.05, ***P<0.001; n.s., not significant. Scale bars: 50 μm.

The transient downregulation of BMP signaling in AT2s early in the regeneration process suggests that the pathway regulates either the proliferation or differentiation of AT2s, or both. To explore these possibilities, we used an ‘alveolosphere’ organoid assay (Barkauskas et al., 2013) in which AT2s, lineage labeled using *Sftpc-CreER^T2^; Rosa26-tdTomato* alleles, are co-cultured in 3D with *Pdgfrα-H2b:GFP^+^* stromal cells, with or without recombinant BMP ligands or antagonists in the medium. We then determined the colony-forming efficiency (CFE) on day 14 post culture by counting the number of spheres >45 μm in diameter (Barkauskas et al., 2013). We observed a significant decrease in CFE in the presence of 20-50 ng/ml BMP4 (Fig. 2A) and a similar effect was seen with BMP2 (Fig. S4). By contrast, there was no significant effect with either BMP5 or BMP6 (Fig. S4A). At both day 7 and 14, the colonies incubated with 50 ng/ml BMP4 were much smaller than controls (Fig. 2A,B). EdU incorporation during a short pulse (2 h before harvest) on day 7 showed that AT2 proliferation is significantly reduced (50%) in the presence of BMP4 compared with controls (Fig. 2B).

Our expression studies indicated that genes encoding the BMP antagonists *Fst* and *Fstl1* are dynamically expressed in regenerating alveolar niche cells. We therefore tested the effect of BMP antagonists in the alveolar organoid system. Analysis of cultures at day 14 indicated no apparent difference in CFE and organoid size (diameter) after treatment with FST, FSTL1 and NOGGIN, compared with controls (Fig. 2C). However, immunohistochemistry of histological sections clearly showed that all three antagonists gave a ∼50% reduction in the percentage of tdTomato^+^ lineage-labeled cells that are HOPX^+^ AT1s (Fig. 2D).

In the course of these studies, we observed that within 2 days of lineage-labeled AT2s being placed in the organoid culture system, the cells co-express both the AT2 marker SFTPC and the AT1 marker AGER (advanced glycosylation end product-specific receptor). AGER was assayed by both immunohistochemistry and by expression of a new *Ager-H2b:Venus* knock-in allele (Fig. S8). Most of the cells remain dual positive at day 7. By day 14, however, AGER^+^ cells, that are also HOPX^+^, are predominantly found in the interior of the spheres with the characteristic elongated morphology of AT1s. By contrast, cuboidal SFTPC^+^ cells are found towards the outside. At day 14, only a small proportion (about 2.6%±0.6%) of the total cells in control spheres are dual positive. By contrast, in the organoids treated with the BMP antagonist FST, not only is the proportion of SFTPC^+^ AT2s increased relative to controls but so too is the proportion of dual positive cells: to 5.5±0.9% of the total (Fig. 2D).

Taken together, our results suggest that in the organoid assay excess BMP ligand negatively regulates AT2 proliferation. By contrast, inhibiting the BMP signaling pathway reduces the differentiation of AT2s to AT1s.

### Enhanced BMPR1a-dependent signaling increases differentiation of AT2s to AT1s in organoids, upregulates AT1 genes and reduces trophic activity of Pdgfrα^+^ fibroblasts

**Fig. 3.**
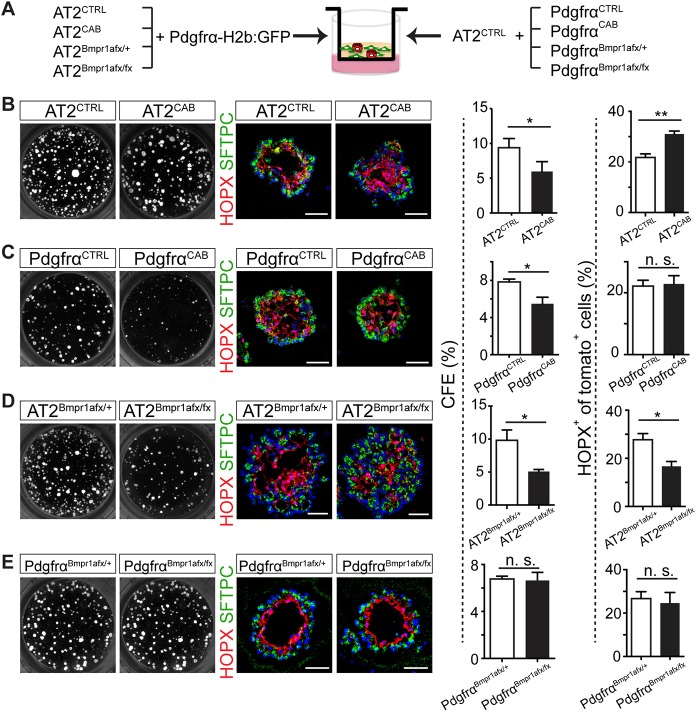
**Effect of BMP pathway activation or inhibition in AT2 or Pdgfrα^+^ cells on AT2 cell behavior in organoid assays.** (A) Schematic of experiments designed to co-culture different classes of AT2s and Pdgfrα^+^ cells in organoid assays. Most cells were also lineage labeled using Rosa26-tdTomato. (B-E) Left: typical organoid cultures and sections immunostained for SFTPC (green) and HOPX (red). Right: quantification of CFEs and percentage of total cells that are HOPX^+^. (B) Constitutively active BMP signaling in AT2s results in lower CFE and enhanced AT1 differentiation. (C) Constitutively active BMP signaling in Pdgfrα^+^ cells reduces CFE but has no effect on AT1 differentiation. (D) Conditional deletion of *Bmpr1a* in AT2s results in reduced CFE and attenuated AT1 differentiation. (E) Conditional deletion of *Bmpr1a* in Pdgfrα^+^ cells has no effects on CFE or differentiation. For all experiments, *n*=3 animals. Data are mean±s.e.m. *P<0.05, **P<0.01; n.s., not significant. Scale bars: 50 μm.

As both AT2 and Pdgfrα^+^ stromal cells express Bmp receptors (Fig. 1D), each cell type has the potential to be affected by exogenous BMP ligands and antagonists in the organoid assay. We therefore used genetic strategies to determine the effect of upregulating or inhibiting BMP signaling in each cell population separately (Fig. 3A). To constitutively activate BMPR1a-dependent signaling in AT2s and to simultaneously lineage trace them, we generated mice with the genotype *Sftpc-CreER^T2^*; *Rosa26-tdTm*/*Rosa26-caBmpr1a* and exposed them to tamoxifen (Tmx). Enhanced BMP signaling in the AT2 population (hereafter AT2^CAB^) was confirmed by qPCR analysis, which indicated significantly increased transcripts of BMP downstream genes, including *Id1*, *Id2* and *Smad6*, compared with control AT2^CTRL^ isolated from mice lacking the *Rosa26-caBmpr1a* allele (Fig. S5A). When AT2^CAB^ were co-cultured with wild-type Pdgfrα^+^ cells, CFE was reduced at 14 days compared with controls (Fig. 3B). It was noted that AT2^CAB^ gave rise to two populations of alveolospheres based on sphere diameters (Fig. 3B). Analysis of pSmad1/5/8 expression suggested that the larger colonies are derived from AT2s that had recombined the *Rosa26-tdTm* but not the *Rosa26-caBmpr1a* allele (Fig. S5D). Significantly, immunofluorescence analysis of the AT2^CAB^ organoids found to have high pSmad1/5/8 signals revealed a 1.5-fold increase in AT1 differentiation compared with AT2^CTRL^ (Fig. 3B and Fig. S5D). To complement these results, we performed RNA sequencing analysis on AT2^CAB^ cells isolated by FACS. As shown in Fig. S6, we found that 119 genes were upregulated more than twofold compared with AT2^CTRL^. Of these genes, 10% are normally preferentially expressed in AT1 cells (Wang et al., 2018; www.lungmap.net). Of the 25 genes downregulated more than twofold in AT2^CAB^ cells, 28% are preferentially expressed in AT2 cells (Fig. S6).

The organoid experiments suggest that upregulation of BMP signaling specifically in AT2s leads to a reduction in both CFE and colony size. However, the values are still higher than in the organoids treated with the highest dose of BMP4 ligand (Fig. 2A). One explanation for this result is that BMP ligand also acts directly on Pdgfrα^+^ stromal cells, reducing their ability to act as trophic support for AT2s. We tested this hypothesis by generating *Pdgfrα-CreER*^*T2*^; *Rosa26-tdTomato/Rosa26-caBmpr1a* mice (hereafter Pdgfrα^CAB^), treating them with Tmx, isolating lineage-labeled Pdgfrα^+^ cells and using them in co-culture assays. As shown in Fig. 3C, the Pdgfrα^CAB^ stromal cells were much less efficient than Pdgfrα^CTRL^ cells in supporting CFE of wild-type AT2s. However, the relative proportion of AT1s to AT2s in the organoids supported by Pdgfrα^CTRL^ or Pdgfrα^CAB^ was the same, as analyzed by SFTPC and HOPX expression (Fig. 3C). Taken together, these results indicate that enhanced BMP signaling can function independently in both AT2s and stromal cells, with the combined effect in organoid assays of reducing the self-renewal of AT2 cells and promoting AT1 differentiation.

### Cell type-specific deletion of *Bmpr1a* reduces differentiation of AT2s to AT1s in the organoid assay

To test the effect of loss of BMPR1a-mediated signaling in AT2s, we isolated lineage-labeled AT2s from *Sftpc-CreER^T2^; Rosa26-tdTomato; Bmpr1a^fx/fx^* mice treated with Tmx (hereafter referred to as AT2^Bmpr1afx/fx^) and co-cultured them with wild-type Pdgfrα^+^ cells. Control studies confirmed that AT2^Bmpr1afx/fx^ cells as a population have reduced expression of transcripts for *Bmpr1a* and downstream target genes (Fig. S5B). As shown in Fig. 3D, reduced signaling through BMPR1a receptor decreased the CFE of AT2^Bmpr1afx/fx^ cells, compared with control AT2^Bmpr1afx/+^ cells, and resulted in attenuated AT1 differentiation. By contrast, when *Bmpr1a* was deleted in Pdgfrα^+^ cells, we observed no difference in either CFE or AT1 differentiation (Fig. 3E).

Together, our data show that either an increase or a decrease in BMP signaling in AT2s results in reduced self-renewal, suggesting that only a narrow range of BMP signaling is effective for AT2 self-renewal in the organoid assay. In addition, BMP signaling in AT2s is required for their efficient differentiation into AT1s.

### *In vivo*, enhanced BMP signaling in Pdgfrα^+^ cells but not in AT2s reduces AT2 proliferation following PNX

**Fig. 4.**
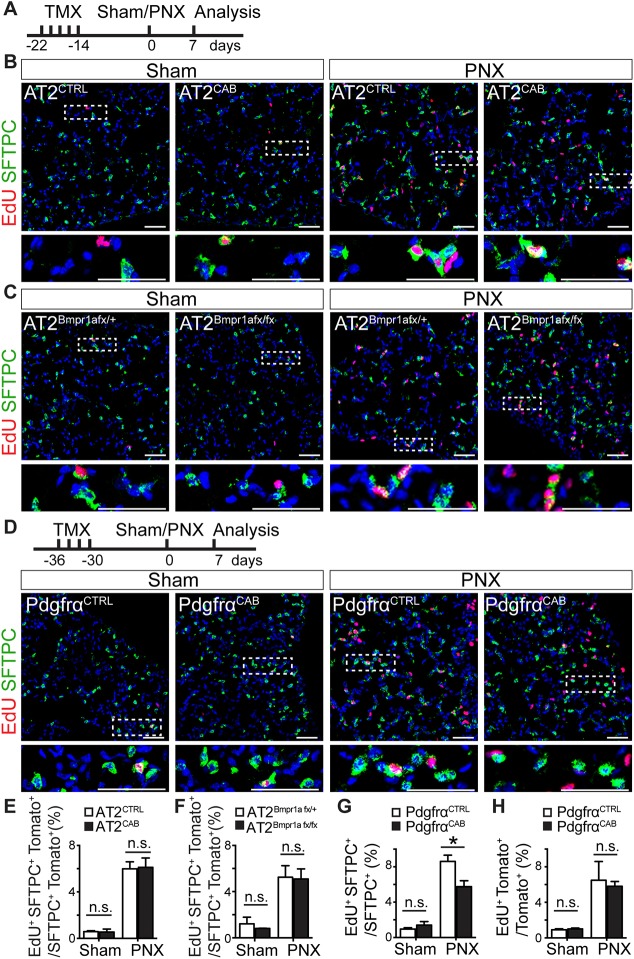
**Manipulating BMP signaling in Pdgfrα^+^ cells, but not in AT2s, reduces AT2 proliferation *in vivo* post-PNX.** (A) Tmx was used to lineage label and conditionally activate or delete Bmpr1a in AT2s or Pdgfrα^+^ cells. After an interval, PNX or sham surgery was performed and 7 days later mice were given a 3 h pulse of EdU. (B-D) Immunofluorescence of lung sections was then used to calculate the percentage of EdU^+^ lineage-labeled SFTPC^+^ AT2s (lineage labeling is not shown) and Pdgfrα^+^ cells. Lower panels are higher magnifications of boxed areas in the panels above. (E,F) Analysis revealed no difference in proliferation between AT2^CAB^ and AT2^CTRL^ cells and AT2^Bmpr1afx/+^ and AT2^Bmpr1afx/fx^ cells. (D,G) By contrast, activation of Bmpr1a in Pdgfrα^+^ cells led to reduced proliferation of AT2s in Pdgfrα^CAB^ versus Pdgfrα^CTRL^ lungs. (H) There is no difference in proliferation of stromal cells in Pdgfrα^CTRL^ and Pdgfrα^CAB^ lungs. For all experiments, *n*≥3 animals/group. Data are mean±s.e.m. n.s., not mentioned. *P<0.05. n.s., not significant. Scale bars: 50 μm.

To examine the effect of gain or loss of BMP signaling on AT2 proliferation during alveolar regrowth *in vivo*, AT2^CTRL^, AT2^CAB^, AT2^Bmpr1afx/+^ and AT2^Bmpr1afx/fx^ mice were pretreated with Tmx 2 weeks before PNX. Given the strong effect of enhanced BMP signaling on the CFE of isolated AT2s *in vitro* (Figs 2A and 3B), we hypothesized that constitutively active BMP signaling in AT2s *in vivo* would lead to a decrease in the number of EdU^+^ AT2s at day 7 post-PNX. Surprisingly, at day 7 post-PNX, the number of EdU^+^ lineage-labeled AT2s was not significantly changed between AT2^CAB^ and AT2^CTRL^ lungs, and in both cases there was a similar increase compared with sham-operated controls (Fig. 4B,E). Likewise, we found no significant differences in the percentage of EdU^+^ lineage-labeled AT2s in the AT2^Bmpr1afx/fx^ lungs compared with AT2^Bmpr1afx/+^ (Fig. 4C,F).

We next asked whether activating BMP signaling in Pdgfrα^+^ cells, by pretreating *Pdgfrα-CreER^T2^; Rosa26-tdTomato/Rosa26-caBmpr1a* mice with Tmx, would have an effect on AT2 proliferation after PNX. In Pdgfrα^CTRL^ mice, 8.6±0.7% of SFTPC^+^ cells were EdU^+^ at 7 days post-PNX. This number was reduced to 5.5±0.6% in Pdgfrα^CAB^ lungs (Fig. 4D,G). Significantly, there was no change in the EdU labeling of Pdgfrα^CAB^ cells compared with Pdgfrα^CTRL^, suggesting that the reduced AT2 proliferation is independent of stromal proliferation (Fig. 4H, Fig. S7).

### BMP signaling in AT2s *in vivo* regulates their differentiation into AT1cells

**Fig. 5.**
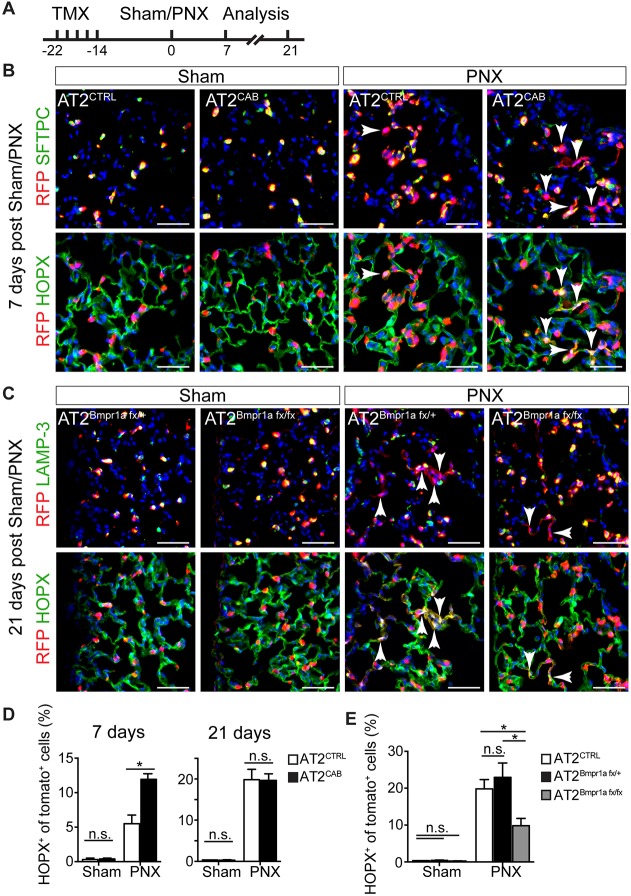
**BMP signaling is required for AT1 differentiation.** (A) Tmx was used to lineage label and conditionally activate or delete Bmpr1a in AT2s. After 14 days, PNX or sham surgery was performed and 7 or 21 days later lungs were harvested and analyzed by immunofluorescence for lineage label (using an antibody to RFP), SFTPC or LAMP-3 (AT2 markers), and HOPX (an AT1 marker). (B) Top panels show expression of lineage label and SFTPC at 7 days post sham surgery or post-PNX in control lungs and lungs in which Bmpr1a was constitutively activated. Bottom panels are the same views exposed to show lineage trace and HOPX. Arrowheads indicate cells losing an AT2 marker and gaining AT1 expression and elongating morphology. (D) Quantification shows that, at 7 days post-PNX, twofold more lineage-labeled AT2^CAB^ cells had given rise to AT1s compared with AT2^CTRL^. However, at 21 days there was no significant difference. (C) Immunohistochemistry 21 days post-PNX of AT2^Bmpr1afx/+^ and AT2^Bmpr1afx/fx^ lungs for RFP (lineage label), LAMP3 and HOPX. (E) Fewer AT1s were derived from AT2^Bmpr1afx/fx^ than from AT2^Bmpr1afx/+^ and AT2^CTRL^. For all experiments, *n*≥3 animals/group. Data are mean±s.e.m. *P<0.05. n.s., not significant. Scale bars: 50 μm.

During alveolar regeneration in response to PNX, AT2s both proliferate and give rise to AT1s (Fig. S3) (Lechner et al., 2017; Liu et al., 2016), the majority of which are pSmad1/5/8 positive at steady state (Fig. 1A). We therefore examined the effects of enhanced BMP signaling on AT2 to AT1 differentiation in AT2^CAB^ versus AT2^CTRL^ lungs after PNX (Fig. 5A). SFTPC and LAMP3, a component of lamellar bodies, were used to mark AT2s, while HOPX identified AT1s. By 7 days post-PNX, 5.5±1.2% of lineage-traced AT2s in control lungs lost expression of an AT2 marker and had differentiated into AT1s, as judged by immunofluorescence analysis for HOPX (Fig. 5B). Significantly, AT2^CAB^ gave rise to more AT1s (11.9±0.9%) at this time (Fig. 5D), a result consistent with our observations in organoid cultures. However, this difference was no longer apparent at day 21 (Fig. 5D).

To further investigate the role of BMP signaling in AT1 differentiation, we then pretreated AT2^Bmpr1afx/+^ and AT2^Bmpr1afx/fx^ mice with Tmx and analyzed lungs 21 days after PNX. While 22.9±3.9% of lineage-labeled AT2^Bmpr1afx/+^ cells gave rise to AT1s (LAMP-3^−^ HOPX^+^), loss of *Bmpr1a* in AT2^Bmpr1afx/fx^ resulted in a significant decrease in AT1 differentiation (9.6±2.7%) (Fig. 5C,E). The phenotype of reduced AT1 differentiation is consistent with the results seen in the organoid assay after treatment with BMP antagonists and downregulating *Bmpr1a* (Figs 2D and 3D).

## DISCUSSION

**Fig. 6.**
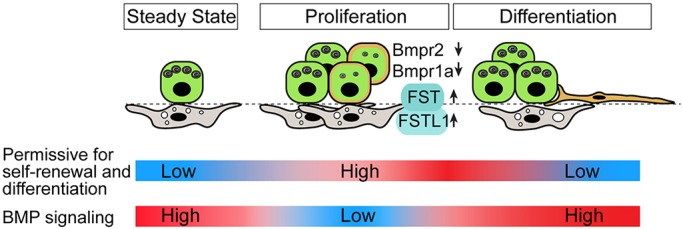
**Schematic model for the role of BMP signaling in the alveolar niche before and after PNX.** At steady state, pSmad1/5/8-dependent signaling is high in the alveolar niche. This helps to maintain the quiescence and identity of AT2s. Following PNX there is a transient decrease in BMP signaling in the lung, with reduced levels of pSmad1/5/8 protein in the nuclei of AT2s and adjacent Pdgfrα^+^ TASCs. This decrease is mediated mostly by changes in the expression of genes encoding BMP receptors and BMP antagonists. We suggest that the reduced BMP signaling primes or permits individual AT2s to be able to respond to trophic and proliferative signals in the niche, including those produced by adjacent TASCs. According to the signals they receive, they may either self-renew or differentiate (e.g. give rise to two AT2s, two AT1s, or one AT2 and one AT1). As remodeling continues, pSmad1/5/8 expression increases in AT2s and this promotes their propensity to differentiate into pSmad1/5/8^+^ AT1s.

A number of studies in mammalian systems have shown that canonical BMP signaling plays complex and differential roles in progenitor cell proliferation and differentiation in tissues such as the epidermis, hair follicles and intestine (Arenkiel et al., 2011; Genander et al., 2014; Haramis et al., 2004; He et al., 2004; Hsu et al., 2014; Lewis et al., 2014). In adult mouse trachea undergoing repair after deletion of luminal cells, BMP signaling inhibits the proliferation of basal stem cells but has no effect on their differentiation into ciliated versus secretory lineages. Moreover, it appears that upregulation of BMP antagonists plays a key role in regulating the stem cell niche (Tadokoro et al., 2016). Here, we present evidence for dynamic changes in BMP signaling during the regrowth of the distal gas-exchange region of the lung following PNX. In this model, formation of new alveoli involves the proliferation and differentiation of AT2s, with about 20% of lineage-labeled AT2 cells generating AT1 cells over 21 days (Fig. S3). In the quiescent adult lung, where there is little cell turnover, immunohistochemistry for nuclear pSmad1/5/8 clearly shows that BMP signaling is active in the majority of AT2s and AT1s, as well as in about half of the Pdgfrα^+^ TASCs located adjacent to AT2s (Fig. 1). Significantly, this active signaling is transiently reduced in both AT2s and TASCs early post-PNX, subsequently returning to previous levels (Fig. 1B,C). This decline appears to be mediated by changes in the transcription of genes encoding some BMP ligands, but significantly also BMP receptors and BMP antagonists (Fig. 1D). Changes in multiple BMP signaling components is a common feature of other examples of tissue remodeling, including the mouse trachea (Hsu et al., 2014; Kosinski et al., 2007; Oshimori and Fuchs, 2012; Tadokoro et al., 2016). Taken together, our findings support the model summarized in Fig. 6. According to this model, BMP signaling in AT2s helps to maintain their quiescence and identity at steady state. A transient decline in pSmad1/5/8 in AT2s early in the post-PNX regrowth phase enables them to transition to a more labile or permissive state in which they can respond to proliferative and differentiation signals. In this state, BMP signaling increases their propensity to differentiate into AT1s.

Initial support for our model came from organoid experiments in which isolated AT2s are grown in 3D in the presence of Pdgfrα^+^ fibroblasts that, together with components of the culture medium, provide trophic support for AT2 self-renewal and differentiation into AT1s. In this assay, the formation of organoids more than 45 μm in diameter depends on a balance between AT2 survival, proliferation and differentiation; signals promoting early AT1 differentiation will likely inhibit CFE as the pool of AT2 cells is quickly exhausted. In organoid cultures, either activating or inhibiting BMP signaling in AT2s using constitutively active *Bmpr1a* or floxed null alleles leads to an attenuated CFE (Fig. 3B,D), indicating that either too much or too little BMP signaling can disrupt colony formation. On the other hand, in those organoids that do reach a scorable size, reduced BMP signaling clearly inhibits AT2 to AT1 differentiation, whereas increased BMP signaling promotes AT1 differentiation.

Given our initial results with organoid assays, it was surprising that manipulation of Bmpr1a-dependent signaling in AT2s *in vivo*, using the same inducible constitutively active *Bmpr1a* allele or *Bmpr1a* floxed null allele, did not apparently affect AT2 proliferation after PNX (Fig. 4). One possible explanation is that the multiple signaling pathways acting *in vivo* after PNX can override or modulate the genetically induced increases or decreases in Bmpr1a signaling, at least in relation to cell proliferation. Such parallel pathways acting in AT2s might include mechanical tension, and factors released by activated macrophages and endothelial cells (Ding et al., 2011; Lechner et al., 2017; Liu et al., 2016; Rafii et al., 2015). Nevertheless, *in vivo* genetic modulation of BMP signaling in AT2s does affect their differentiation; loss of Bmpr1a in cells homozygous for the floxed alleles results in fewer AT1s, whereas activation of signaling in AT2^CAB^ cells accelerates their differentiation into AT1s at 7 days post-PNX (Fig. 5B,D). The fact that no excess conversion of AT2s to AT1s is seen at 21 days suggests that only a fixed level of conversion is needed to restore lung homeostasis after PNX.

In the future, we need to know more about the mechanisms by which BMP signaling enhances the propensity of AT2s to differentiate into AT1s and how the BMP pathway interacts with other pathways affecting self-renewal versus differentiation. Recent experiments have indicated that canonical WNT signaling in AT2s inhibits AT1 fate choice (Frank et al., 2016; Nabhan et al., 2018), pointing to opposing effects between the two pathways. Such antagonistic BMP/WNT effects have been described in various other progenitors and stem cell niches, including intestinal stem cells, hair follicles and cardiomyoblasts (Genander et al., 2014; Jain et al., 2015b; Takeda et al., 2011). Studies with AT2s in culture have also suggested that there is antagonism between BMP and TGFβ signaling, with the latter promoting AT1 differentiation (Zhao et al., 2013). Although our results with BMP signaling do not support their model, it is of interest that AT2^CAB^ cells upregulate *Tgfb1* expression about twofold, along with several genes that are expressed in AT1 cells. Finally, during cardiomyogenesis, active pSmad1/5/8 complexes physically interact with HOPX to repress the WNT pathway (Jain et al., 2015b). The fact that HOPX is highly enriched in AT1s raises the possibility that BMP drives AT1 differentiation through HOPX-mediated mechanisms.

One new observation reported here is that soon after lineage-labeled AT2 cells are placed in 3D culture, they co-express genes typically associated with AT2s (*Sftpc*) and AT1s (*Ager*), and this co-expression continues for up to 10 days (Fig. S8). We speculate that this phenotype is associated with the ‘priming’ or increased plasticity of AT2s proposed in our model (Fig. 6). *In vivo*, at steady state, fewer than 1% of AT2 cells are dual positive, but this value increases to 20% at day 7 post-PNX (Fig. S8). Future studies will need to test the functional significance of the dual positive phenotype.

Finally, another novel finding from our studies both *in vitro* and *in vivo* is that constitutive activation of BMP signaling in Pdgfrα^+^ mesenchymal cells inhibits their ability to support AT2 self-renewal both *in vivo* and *in vitro* (Figs 3C and 4D). This likely contributes to the lower CFE of AT2s in the organoid assay in the presence of BMP4 ligand (Figs 2A and 3C). Recent studies have presented evidence that *in vivo* there are at least two populations of stromal cells in the alveolar niche, only one of which, mesenchymal alveolar niche cells (MANCs), promotes *in vitro* alveolar organoid growth (Zepp et al., 2017). Using single-cell RNA-seq analysis, Zepp et al. identified a BMP antagonist, *Grem2*, as one of the regulators secreted by MANCs but not by other mesenchymal populations. By contrast, our data show that the transcription of *Grem2* does not change in Pdgfrα^+^ stromal cells post-PNX (Fig. 1D), but rather indicate that FSTL1 and FST are the major antagonists regulating BMP signaling, at least in the PNX model. Further experiments are required to localize transcripts and protein for the multiple BMP ligands and antagonists at the single-cell level in alveoli at different times during the regrowth process. In addition, it will be important to clarify whether the Axin2^+^ stromal cells identified by Zepp et al. are identical to the Pdgfrα^+^ cells adjacent to AT2s that we have here termed TASCs. These cells have a very characteristic morphology, with extended processes (Fig. S1 and Movie 1). Fibroblasts that appear to have a similar morphology and to make contact with AT2s, AT1 and endothelial cells have been described in the human lung (Sirianni et al., 2003).

## MATERIALS AND METHODS

### Mice

To generate *Ager-H2b:Venus* mice, a DNA fragment containing 8 kb upstream of the first coding exon and exons 2-7 were retrieved from a BAC clone (bMQ174, Source BioScience) and recombined into the vector pL25B upstream of a HSV-TK cassette for negative selection. A cassette encoding H2b:Venus fusion protein followed by polyA (kindly provided by Dr Anna-Katerina Hadjantonakis, Sloan Kettering Cancer Center) and a neo cassette flanked with FRT sites were recombined into the start codon. The construct was electroporated into G4 (C57BL/6Ncr×129S6/SvEvTac) hybrid ES cells. Two clones were injected into C57BL/6 blastocysts. Mice were bred to 129S4-Gt(ROSA)26Sor^tm2(FLP*)Sor^/J to remove the neo cassette. *Ager-H2b:Venus* mice are maintained on a C57BL/6 background.

A similar strategy was used to generate the *Pdgfrα-CreER^T2^* ‘knock-in’ allele. A CreER^T2^ poly-A cassette and a FRT-flanked neo cassette were recombined into the start codon of Pdgfrα (BAC clone: bMQ123p11, Source BioScience). The construct was electroporated into TL1 (129S6/SvEvTac) ES cells and these were injected into C57BL/6 blastocysts. The neo cassette was removed. *Pdgfrα-CreER^T2^* mice were maintained on a C57BL/6 background.

*Sftpctm1(cre/ERT)Blh* (*Sftpc-CreER^T2^*) (Rock et al., 2011), *Rosa26-CAG-lsl-tdTomato* (Arenkiel et al., 2011), *Rosa26-CAG-lsl-caBmpr1a* (Rodriguez et al., 2010), *Bmpr1a flox* (Mishina et al., 2002) and *Pdgfra^tm11(EGFP)Sor^* (*Pdgfra-H2b:GFP*) (Hamilton et al., 2003) were maintained on a C57BL/6 background. All experiments were performed according to IACUC-approved protocols.

### Pneumonectomy

Procedures were performed as previously described (Lechner et al., 2017). Briefly, mice were anesthetized with 2% isofluorane and intubated using a Harvard mini-vent ventilator with 200 µl stroke volume at 200 strokes per minute. The left pulmonary vasculature and bronchus were ligated with a titanium clip and the left lobe was removed. After closing the ribs, an angiocath was inserted to remove air to re-establish negative pressure. Mice were disconnected from the ventilator when autonomous breathing recovered. Sham animals underwent the same procedures without removing the left lobe.

### Lung dissociation and FACS

Lungs were inflated intratracheally with 1-1.5 ml of protease solution containing collagenase type I (450 U/ml; Gibco #17100-017), elastase (4 U/ml; Worthington Biochemical Corporation #LS002279), dispase (5 U/ml; BD Biosciences #354235) and DNaseI (0.33 U/ml) in DMEM/F12. Lung lobes were separated, cut into small pieces and incubated with 3 ml protease solution for 30 min at 37°C with frequent agitation. Equal amounts of media containing 10% FBS was added to the tissue suspension and then filtered through a 100 µm strainer. The cell pellet was then resuspended and incubated with 2 ml of red blood cell lysis buffer (eBioscience) for 2 min at room temperature. The cell suspension was washed with 10% FBS, filtered through a 40 µm strainer, centrifuged and resuspended in DMEM/F12+2% BSA. Sorting was performed using a FACS Vantage SE.

### Organoid culture

FACS sorted cells were resuspended in MTEC/Plus media and mixed 1:1 with growth factor-reduced Matrigel (BD Biosciences #356230). MTEC/Plus:Matrigel (90 µl) containing 5×10^3^ AT2s and 5×10^4^ stromal cells was seeded in individual 24-well 0.4 µm Transwell inserts (Falcon). MTEC/Plus (500 µl) was placed in the lower chamber and media was changed every other day. Spheres were counted and fixed on day 14. Recombinant proteins were purchased from R&D systems and used as follows: BMP2 (50 ng/ml), BMP4 (50 ng/ml), FST (500 ng/ml), FSTL1 (500 ng/ml) and Noggin (1000 ng/ml). Each condition was tested in at least three wells and each experiment was repeated at least three times.

### Histology and immunofluorescence analysis

Lungs were inflated with 4% paraformaldehyde to 25 cm H_2_O pressure for 10 min and then removed and submerged in 4% paraformaldehyde in PBS for 4 h at 4°C. For tissues used for phospho-Smad1/5/8 detection, PhosStop (Roche 4906845001) was added to the fixative. Tissue was dehydrated, embedded in paraffin and sectioned at 7 µm. Tissue sections underwent 10 mM sodium citrate antigen retrieval and were blocked with 3% BSA, 10% donkey serum and 0.1% Triton X-100 for 1 h at room temperature. Primary antibodies diluted in block were applied and incubated overnight at 4°C. Tissue sections were washed with PBS and fluorophore-conjugated secondary antibodies were diluted at 1:500 and incubated for 1 h at room temperature. For phospho-Smad1/5/8 staining, HRP-conjugated secondary antibody (1:1000) and TSA detection system were used (PerkinElmer, NEL744001KT). Primary antibodies were as follows: LAMP-3/CD208 (Dendritics, DDX0191, 1:200), endomucin (Santa Cruz, sc-65495, 1:250), GFP (Aves lab, GFP-1020, 1:500), HOPX (Santa Cruz, sc-398703, 1:50), RFP (Rockland, 600401379, 1:250), PDGFRβ (Cell Signaling, 3169, 1:100), RAGE/AGER (R&D, MAB1179, 1:200), SFTPC (Millipore, ab3786, 1:500; Santa Cruz, SC-7706, 1:100), phospho-Smad1/5/8 (Millipore, AB3848-I, 1:250; Cell Signaling, 9511). Images were obtained using Zeiss LSM 710, LSM 780 and Imager AxioCam microscopes.

### Quantification and statistics

For quantification, two well-separated longitudinal sections per accessary lobe were imaged and the whole areas were analyzed using ImageJ. *n*≥3 animals/experiment. Sections of organoids (≥10 organoids/transwell) were analyzed after immunohistochemistry. The quantification values of triplicate wells were averaged and plotted using Prism software. Statistical analysis was performed using unpaired, two-tailed, Student's *t*-test between groups. Values on graphs are shown as mean±s.e.m.

### Quantitative RT-PCR

Total RNA was extracted from FACS-sorted lineage-labeled AT2s and Pdgfra-H2b:GFP cells using Direct-zol RNA MiniPrep Kit (Zymo Research). cDNA was synthesized using SuperScript VILO kit (Invitrogen). qPCR was performed with iQ SYBR Green Supermix (Bio-Rad) and StepOne Plus system (Applied Biosystems). The mRNA levels of target genes were normalized to *Gapdh*. Primer sequences are in Table S1.

### RNA sequencing and analysis

Total RNA was extracted from FACS-sorted lineage-labeled AT2^CTRL^ and AT2^CAB^ cells using Direct-zol RNA MiniPrep Kit (Zymo Research) and mRNA was enriched from 200 ng of each total RNA using NEBNext Poly(A) mRNA Magnetic Isolation Module (New England BioLabs). Libraries were prepared using NEBNext Ultra II RNA Library Prep Kit for Illumina (New England BioLabs). Paired-end sequencing (150 bp for each read) was performed using HiSeq X with the depth of 24 million reads for each sample. The quality of sequenced reads was assessed using FastQC (www.bioinformatics.babraham.ac.uk/projects/fastqc/). PolyA/T tails were trimmed using PRINSEQ (Schmieder and Edwards, 2011). Adaptor sequences were trimmed and shorter reads than 24 bp were dropped using Trimmomatic (Bolger et al., 2014). Reads were mapped to the mouse reference genome (mm10) using Hisat2 (Kim et al., 2015) with default setting. Duplicate reads were removed using the markdup option of SAMtools (Li et al., 2009). Fragment numbers were counted using the featureCounts option of SUBREAD (Liao et al., 2014). Normalization and extraction of differentially expressed genes (DEGs) between AT2^CTRL^ and AT2^CAB^ were performed using an R package, DESeq2 (Love et al., 2014). Heatmaps were generated using Shinyheatmap (Khomtchouk et al., 2017). The RNA-seq data have been deposited in GEO under accession number GSE112431.

### Western blot

Protein extracts were collected from accessory lobes. Equal amounts of proteins were separated by SDS-PAGE and transferred to polyvinylidene fluoride membranes. Membranes were blocked for 1 h with 5% BSA in TBST (0.1% Tween 20) and then incubated with phospho-Smad1/5/8 antibody (Cell Signaling, 13820, 1:1000) and β-actin antibody (Abcam, ab8226, 1:2000) overnight at 4°C. Membranes were washed with TBST and then incubated with HRP-conjugated secondary antibodies (Jackson ImmunoResearch, 711-035-152, 715-035-151 1:10,000). Protein blots were analyzed with the ECL detection system (FEMTOMAX-110, Rockland Immunochemicals).

## Supplementary Material

Supplementary information
