# Supplementary material for: Niche-mediated BMP/SMAD signaling regulates lung alveolar stem cell proliferation and differentiation
Source: Development. 2018 May 11;145(9):dev163014. doi: 10.1242/dev.163014 (PMC5992594; doi:10.1242/dev.163014)
Supplement: Supplementary information [file develop-145-163014-s1.pdf]

A

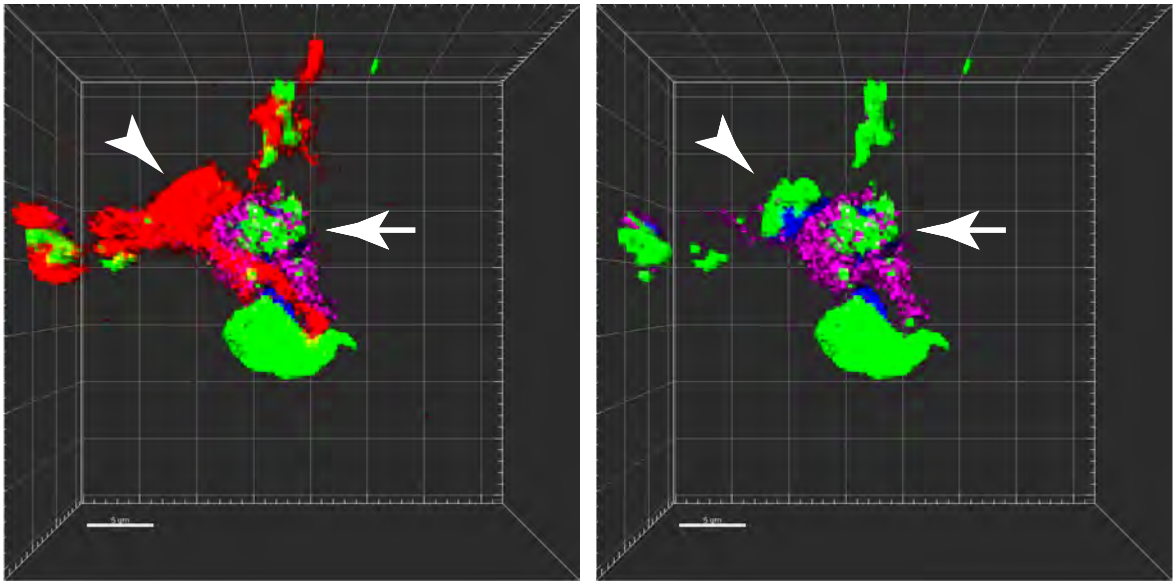

RFP pSmad1/5/8 LAMP-3

B

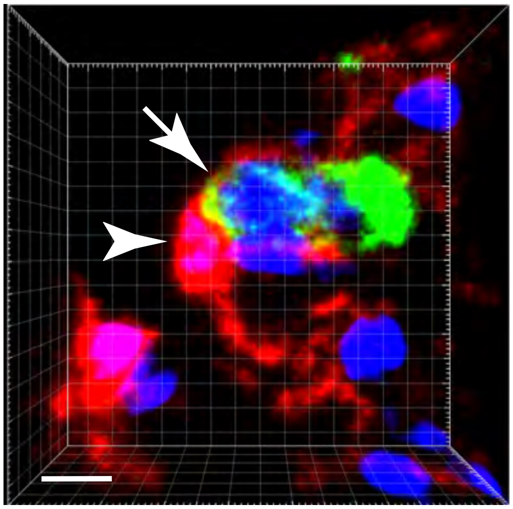

RFP Lamp-3

Fig. S1. Cellular Morphology of TASCs (Type 2 - associated stromal cells)
(A) *Pdgfrα-CreER^{T2}; Rosa26-tdTomato* mice were treated with Tmx and lungs were fixed 4 days later. Images show that a representative Pdgfrα+ cell (RFP antibody, arrowhead) closely adjacent to an AT2 (LAMP-3^{+}, magenta, arrow) has long cellular extensions. Both cells in the niche are pSmad1/5/8^{+} (green) (B) A second example of a Pdgfrα^{+} lineage labeled TASC (arrowhead) adjacent to AT2 cells (arrow). 3D rotation shown in movie 1.

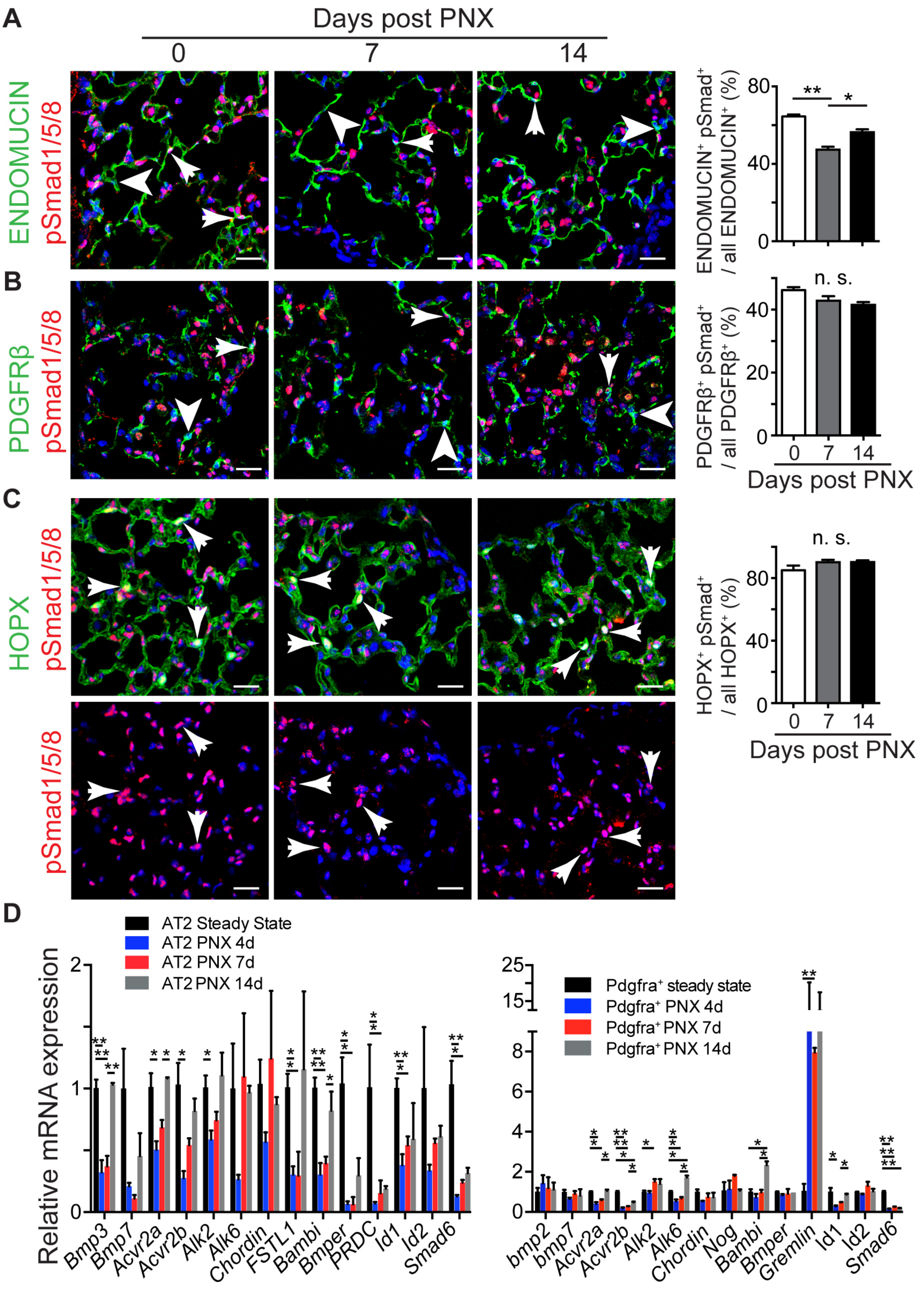

Development • Supplementary information

Fig. S2. BMP signaling during alveolar regrowth *in vivo*

Expression of pSmad1/5/8 (red) in (A) Endomucin$^+$ (B) PDGFRβ$^+$ and (C) HOPX$^+$ cells at steady state and after PNX. Only nuclei that could be unambiguously assigned to a specific cell were scored as either positive for pSmad (arrows) or negative (arrowhead). Scale bars 20 μm. (D) qPCR for BMP signaling genes in AT2s and Pdgfrα$^+$ cells at steady state and times after PNX. The mRNA expression levels of PNX samples were normalized to steady state levels. Data shown are mean ± SEM. *p < 0.05, **p < 0.01.

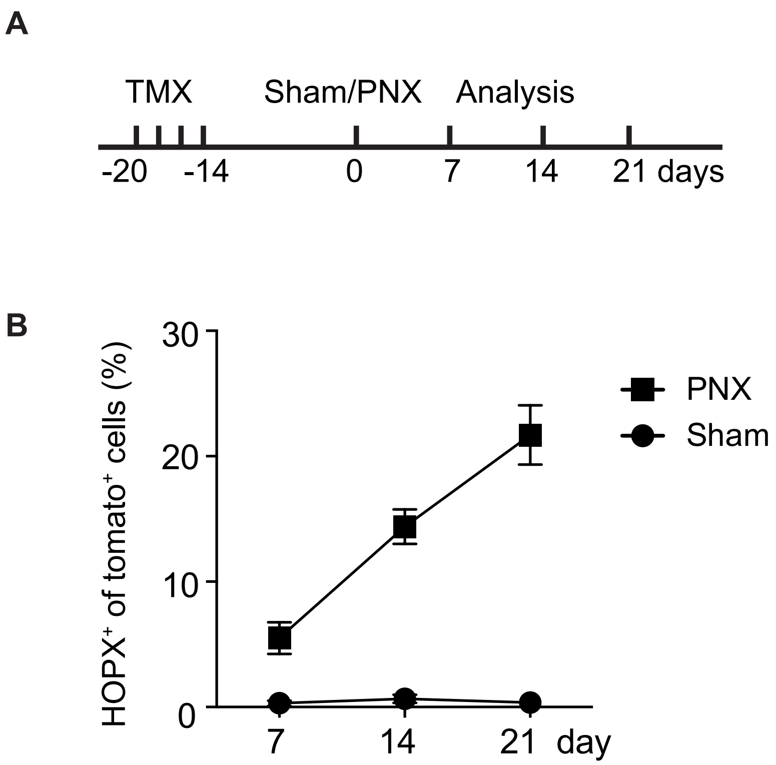

Fig. S3. AT2 differentiation into AT1s during alveolar regrowth

(A) *Sftpc-CreER^{T2};* Rosa26-tdTomato mice were treated with Tmx two weeks before sham surgery or PNX and lungs fixed 7d, 14d, and 21d later. (B) Number of lineage labeled AT2-derived AT1s was determined using RFP and HOPX immunofluorescence as shown in Fig.5. n ≥ 3 animals/group.

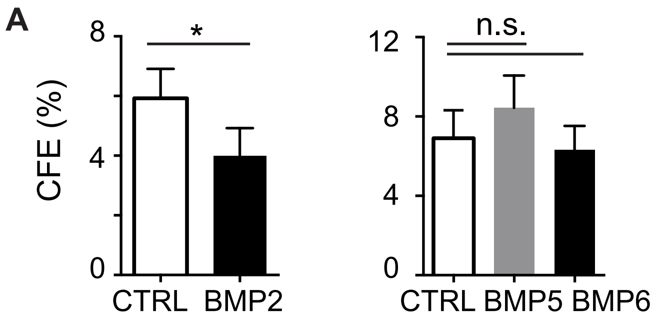

Fig. S4. Effect of BMP ligands on AT2 organoid culture

(A) AT2 organoid culture in the presence of BMP ligands (all 50 ng/ml). While adding BMP2 reduces CFE, no effect was seen with BMP5 and BMP6.

Development • Supplementary information

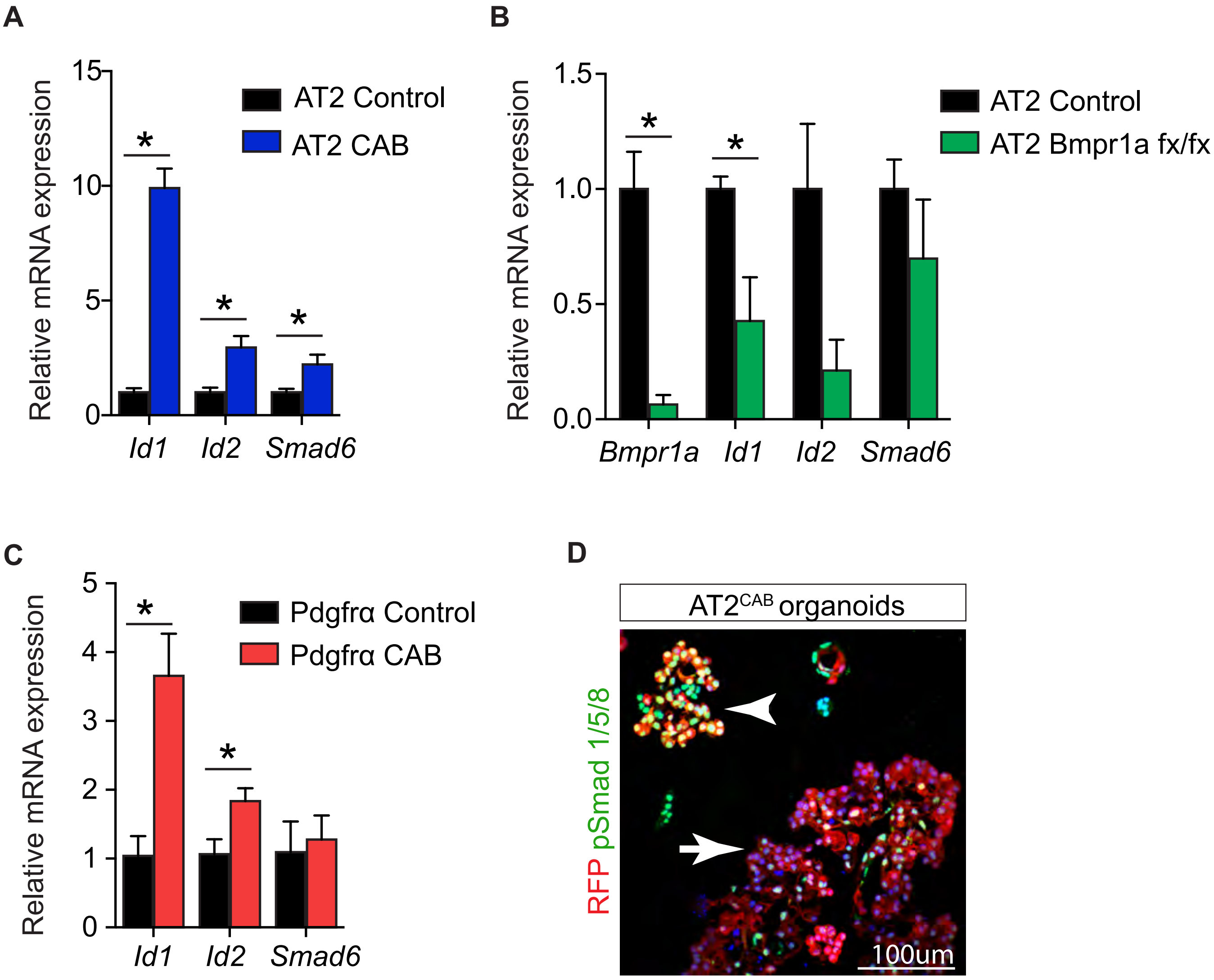

Fig. S5. Validation of enhanced and inhibited BMP signaling

qPCR analysis for BMP downstream targets in (A) $AT2^{CTRL}$ and $AT2^{CAB}$, (B) $AT2^{CTRL}$ and $AT2^{Bmpr1a\ fx/fx}$ and (C) $Pdgfr\alpha^{CTRL}$ and $Pdgfr\alpha^{CAB}$ cells. Data shown are mean ± SEM. *$p < 0.05$. (D) Typical high pSmad1/5/8 expression in a lineage labeled $AT2^{CAB}$-derived organoid (arrowhead). Levels were typically lower in the few large organoids (arrow), suggesting these were derived from AT2s that had recombined the Rosa26-tdTomato but not the Rosa26-caBmpr1a allele. n = 3 animals/group. Data shown as mean ± SEM. Scale bar 100 µm.

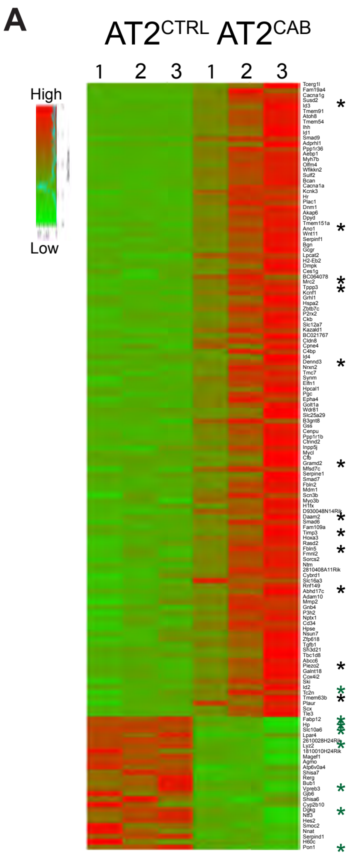

Fig. S6. RNA sequencing analysis of AT2^{CTRL} and AT2^{CAB} Heatmap showing 119 genes upregulated more than two-fold in AT2^{CAB} compared with AT2^{CTRL}. Of these genes, 12 are normally preferentially expressed in AT1 cells (black star). Of the 25 genes preferentially downregulated more than 2 fold in AT2^{CAB} cells, 8 are preferentially expressed in AT2 cells (green star). P-adj < 0.05.

Development • Supplementary information

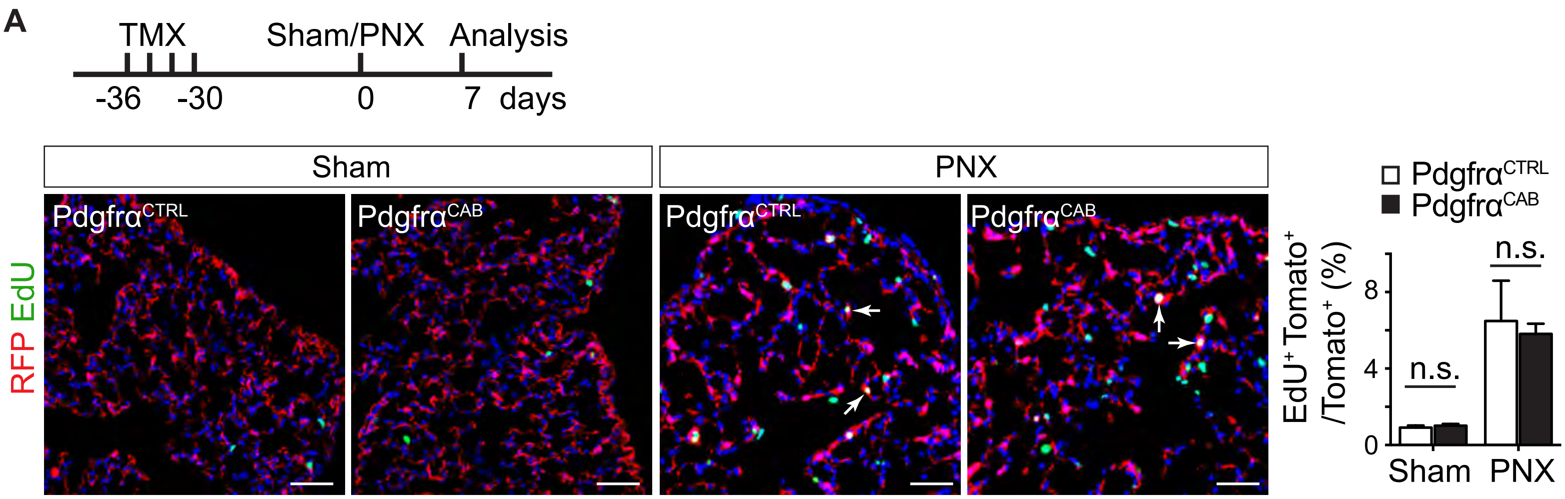

Fig. S7. Enhanced BMP signaling in Pdgfrα stromal cells has no effect on their proliferation

(A) Tmx was used to lineage label and conditionally activate Bmpr1a in Pdgfrα^{+} cells. Pdgfrα^{CTRL} and Pdgfrα^{CAB} lungs were fixed 7 days post sham or PNX surgery. Immunofluorescence analysis of RFP (lineage label) and EdU showed no significant differences of proliferation in stromal cells of Pdgfrα^{CTRL} and Pdgfrα^{CAB} lungs. n ≥ 3 animals/group. Data shown as mean ± SEM. Scale bars 50 μm.

Development • Supplementary information

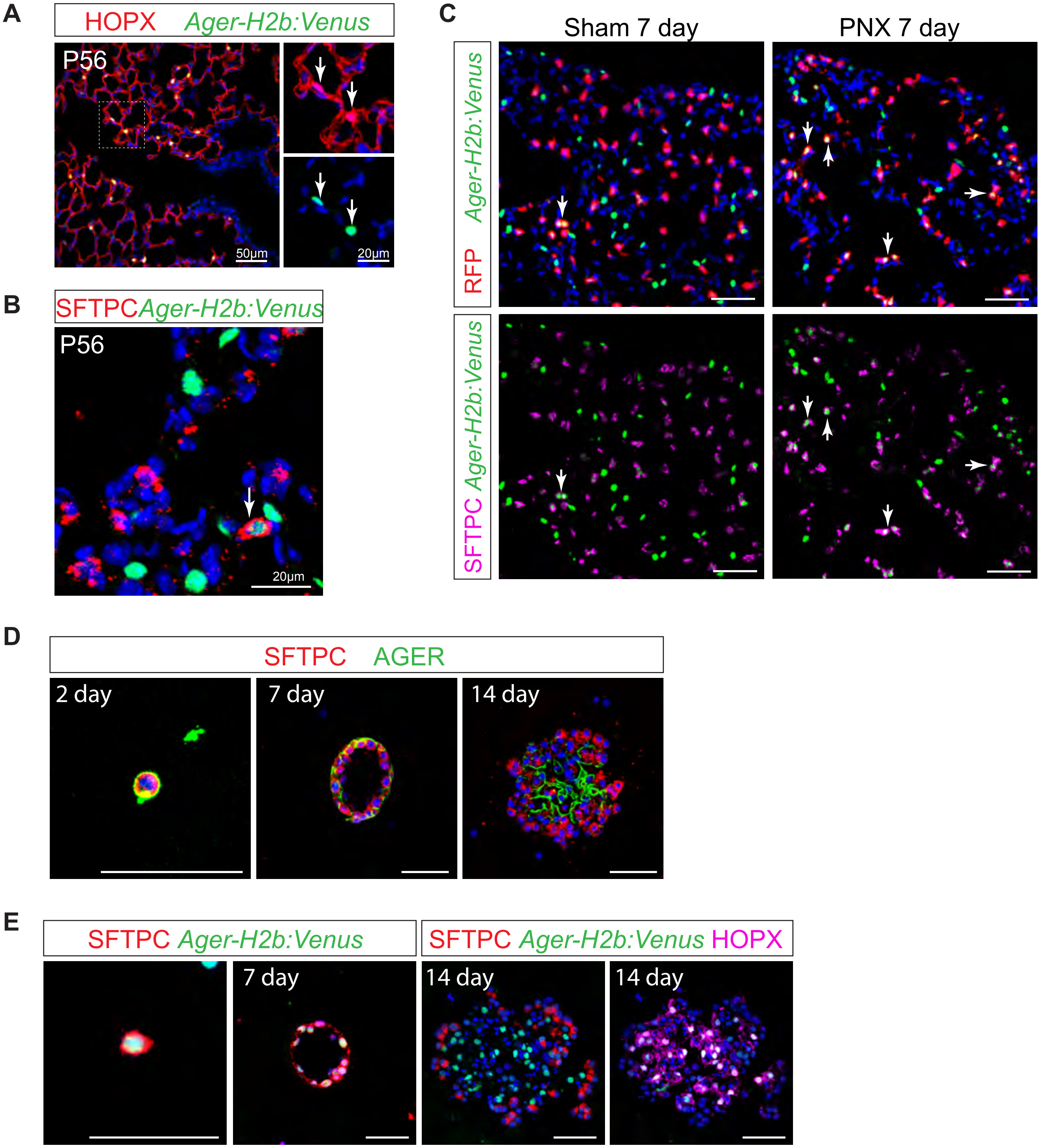
A
HOPX Ager-H2b:Venus
P56
50µm
20µm
B
SFTPC Ager-H2b:Venus
P56
20µm
C
Sham 7 day
PNX 7 day
RFP Ager-H2b:Venus
SFTPC Ager-H2b:Venus
D
SFTPC AGER
2 day
7 day
14 day
E
SFTPC Ager-H2b:Venus
SFTPC Ager-H2b:Venus HOPX
7 day
14 day
14 day

Fig. S8. SFTPC and AGER dual positive cells in regeneration systems including PNX and organoid cultures

(A) Evidence that a new knock-in allele, *Ager-H2b:Venus*, labels AT1s, as judged by co-expression (using antibodies to GFP) with HOPX in nuclei. (B) Note that < 1% of SFTPC^{+} cells express a high *Ager* signal (arrow) at steady state (P56). (C) Tmx treated *Sftpc-CreER^{T2}; Rosa26-tdTomato; Ager-H2b:Venus* mice were analyzed 7 days post sham and PNX surgery. Arrows indicate lineage labeled (RFP^{+}) AT2s that are SFTPC^{+} Ager^{+}. The number of these double positive cells increases in the post-PNX lung (20%), compared to sham (2.5%) and steady state (< 1%). (D) Analysis of sections of AT2 cultures using antibodies for SFTPC, AGER, H2B:Venus (GFP) and HOPX. Nearly all cells are SFTPC^{+} AGER^{+} double positive at 2 and 7 days while the number is reduced at 14 days. At 14 day, *Ager*$^{+}$ HOPX^{+} AT1s are found internal to the sphere while SFTPC^{+} AT2s are located towards the periphery. Scale bars 50 µm.

Development • Supplementary information

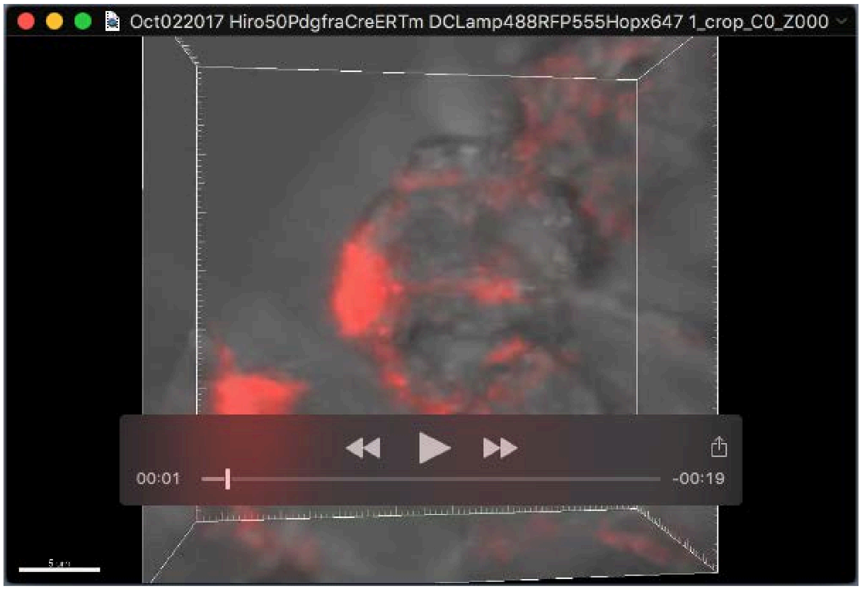

Movie 1. 3D rotation of AT2-TASC

Long cellular extensions of a TASC (red) closely adjacent to an AT2 (green).

Development • Supplementary information

Table S1. Primer sequences

Primers	forward	reverse
qPCR		
Bmp2	5'-CAG AAG CCC AGT TGC TGC TC-3'	5'-AAC ACT AGA AGA CAG CGG GTC C-3'
Bmp4	5'-GGA ACA GGG CTT CCA CCG TA-3'	5'-ACT GCA GGG CTC ACA TCG AA-3'
Bmp5	5'-GGG TAT TGT GGG CTT CCT CT-3'	5'-TTT CCC GTC TCT CAT GGT TC-3'
Bmp6	5'-CAG AGT CGC AAC CGG TCC AC-3'	5'-TGC AAT GAT CCA GTC CTG CCA-3'
Bmp7	5'-CAA GAC GCC AAA GAA CCA AG-3'	5'-CAG CTC ATG TTT CTT GCA GG-3'
Bmpr2	5'-CCT TGA CCT GGA TAA CCT GAA G-3'	5'-TTA CAG CAA CTG GAC GCT C-3'
Bmpr1a	5'-CCT GAT TGA CCA GTC CCA AA-3'	5'-CTT TAC CAA CCT GCC GAA CC-3'
Bmpr1b	5'-ACT CTA CGG TAC TGC AGG GC-3'	5'-GCG CTT CGA TGG CTT AGC TT-3'
Fst	5'-CAA GGT TGG CAG AGG TCG CT-3'	5'-AGA AGC ACG CCA GAA GAG CA-3'
Fstl1	5'-AGC CAT CAA CAT CAC CAC TTA T-3'	5'-AGT TTC CAG TCA GCG TTC TC-3'
Gremlin	5'-AAG CGA GAT TGG TGC AAA AC-3'	5'-TGA AAG GAC CCT TCC TCC TT-3'
Gremlin2	5'-GGA TGT TCT GGA AGC TCT CG-3'	5'-GAT CTG GTG ATG CCA CCT CT-3'
Noggin	5'-ACG TCA AAA GTT ACC CGG CTT-3'	5'-CCA TGC GAA GGG TAC TGG GA-3'
Chordin	5'-CGA GGG GCT CAT ATG CTG CT-3'	5'-GGC AAT CTG TCA TAG CGG GC-3'
Acvr2a	5'-GCG TTC GCC GTC TTT CTT ATC-3'	5'-GTT GGT TCT GTC TCT TTC CCA AT-3'
Acvr2b	5'-GGC CAT GTA CCG TCT GGT-3'	5'-TGG CTG TTC GGT TTG AGC-3'
Acvr1(Alk2)	5'-CAT CAG GAA GTG GCT CCG GT-3'	5'-TCC CGA CAC ACT CCA ACA GG-3'
Bambi	5'-CTG TGA TAG CGG TTC CCA TT-3'	5'-TGG TGT CCG TGA AAG CTG TA-3'
Bmper	5'-GGT GAC TTG TAA GAG GGA GAA G-3'	5'-GTT TGC CAC TTG AAG GAA CTG-3'
Id1	5'-GGC GAG ATC AGT GCC TTG-3'	5'-AAG GGC TGG AGT CCA TCT G-3'
Id2	5'-ATG AAA GCC TTC AGT CCG GTG-3'	5'-AGC AGA CTC ATC GGG TCG T-3'
Smad6	5'-AAT TCT CAG ATG CCA GCA TG-3'	5'-AGG TAG GTC GTA GAA GAT GC-3'

Development • Supplementary information
